# Effect of two (short-term) storage methods on load to failure testing of murine bone tissue

**DOI:** 10.1038/s41598-019-42476-4

**Published:** 2019-04-11

**Authors:** Thomas M. Tiefenboeck, Stephan Payr, Olga Bajenov, Thomas Koch, Micha Komjati, Kambiz Sarahrudi

**Affiliations:** 1 Department of Orthopaedics and Trauma Surgery, Division of Trauma Surgery, Medical University of Vienna, Vienna, Austria; 2 Institute of Materials Science and Technology, TU Wien, Vienna, Austria; 3 Department of Orthopaedics, Sacred Heart Hospital of Vienna, Vienna, Austria

## Abstract

Since mechanical testing of bone quality is often delayed following euthanasia, the method of bone storage is of high importance in animal studies. Different storage methods may cause a change in the properties of bone tissue during mechanical testing. Therefore, the aim of this study was to investigate the biomechanical effects of two different fixation methods for bone tissue. We hypothesized that there is a difference between the load to failure values between the two groups. The tibias of fifteen 18-week-old female C57BL/6 mice were harvested and randomly allocated to three different groups with varying storage methods: (1) frozen at −80 °C, (2) paraformaldehyde working solution, and (3) native group. A storage time of two weeks prior to testing was chosen for groups 1 and 2. In group 3, referred to as the “native group”, bones were immediately tested after the harvesting procedure. The comparison of the mean load to failure of all 3 groups (group 1: 28.7 N ± 6.1 N, group 2: 23.8 N ± 3.8 N and group 3: 23.7 N ± 5.7 N) did not reveal a significant difference. There was also no difference in strength or stiffness. The findings of the present study demonstrate that the two most common storage methods, do not have an influence on the biomechanical properties of murine bone over a two week period.

## Introduction

In the past few years the mechanical measurements of bone tissue, especially in small animal models, have increasingly gained importance^1,2^. Mouse bone has proven to be excellent for mechanical testing^1^. The analysis of the mechanical properties of the bone is indispensable for experimental studies on bone, especially in the field of trauma and orthopaedics^3^. Stiffness, strength and loading capacity can only be evaluated by mechanical testing^4^. Normally, the widely used load to failure test is performed as the final procedure and is usually reserved for a latter stage of the experiment. Therefore, it is necessary to store bone for a longer time period from harvesting to testing^3^. Several different techniques of bone storage, such as freezing or fixation with varying methods, for example, with ethanol, formaldehyde or paraformaldehyde, have been described in current literature. All of these techniques carry certain advantages and disadvantages^5,6^. For bone freezing, different protocols varying from −80 °C to −4 °C exist. In most cases, the bone is frozen to −20° and rehydrated to regain normal mechanical properties for testing^7,8^.

Regarding formalin and ethanol based storage methods, negative effects are described in the literature. Formalin based methods reveal changes of the organic matrix with irreversible alteration of material and mechanical properties caused by formalin, especially after storage periods over eight weeks^9,10^. Even immediate impairment of biomechanical properties is described when using ethanol^9^. However, an advantage of these storage methods is that they simultaneously resemble fixation methods, which are necessary when further histological investigation is planned.

In order to obtain valid data it is of paramount importance to understand whether or not different storage methods affect bone properties, such as stiffness and strength. Thus, the aim of this study was to compare the influence of two different preservation methods on mechanical properties of murine bone tissue by using the generally accepted load to failure test. We hypothesized that paraformaldehyde and freezing have different effects on the mechanical properties (load to failure, strength and stiffness) of murine bone.

## Results

The mean overall weight of the mice was 25.3 g (range; 21.9 g to 29 g; median 25 g). The mean bodyweight of the animals in group 1 was 25.6 ± 1.13 g (median; 24.9; range 24.5 to 27.3 g). The animals in group 2 weighed 24.2 ± 1.49 g (median; 24.4; range 21.9 to 25.9 gram) and 25.9 ± 1.9 g (median; 25.6; range 23.5 to 29.1 g) in group 3. There was no difference in mean weight between all groups.

The mean length of harvested tibias was 18 mm (range; 18 to 20 mm, median 18 mm, STD 0.68 mm); the mean diameter of the ROI was 1.51 mm (range; 1.2 – to 1.85 mm, median 1.49 mm, STD 0.18 mm). The mean cross sectional area of ROI (fracture region) was 1.81 mm^2^ (median; 1.74 mm^2^ range; 1.13 to 2.69 mm^2^ STD 0.44 mm^2^). There was no significant difference between mean stiffness (freezing 37.5 N/mm^2^ vs. native 39.2 N/mm^2^ vs. 32.7 N/mm^2^ paraformaldehyde) and strength (freezing 17.6 N/mm^2^ vs. native 14.3 N/mm^2^ vs. 13.94 N/mm^2^ paraformaldehyde). A detailed overview is presented in Table 1.

**Table 1:** Detailed overview of stiffness, strength, cross sectional area and load to failure of tested bone.

Nr. Group	Stiffness in N/mm^2^	Strength in N/mm^2^	Cross sectional area in mm^2^	Load to failure in N
**Freezing (n** = **12)**				
1	44.6	16.8	1.23	20.6
2	36.8	16.6	1.21	20
3	44.8	21.0	1.91	40.2
4	39.0	15.4	1.63	25
5	52.8	12.7	1.89	23.9
6	27.1	16.2	1.74	28.2
7	19.8	−27.1	1.13	30.6
8	23.5	15.1	1.67	25.2
9	28.3	19.1	1.43	27.4
10	27.2	12.6	2.57	32.3
11	42.6	18.3	1.79	32.7
12	63.4	20.9	1.84	38.5
**Native Bone (n** = **9)**				
13	49.6	11.3	2.52	28.4
14	44.3	10.4	1.52	15.8
15	29.3	10.6	2.01	21.4
16	39.7	12.0	2.66	18.1
17	32.0	23.1	1.33	30.7
18	48.2	12.1	2.69	17.2
19	24.9	13.3	1.72	22.8
20	38.4	18.7	1.41	26.3
21	46.3	13.9	2.32	32.2
**Paraformaldehyde (n** = **8)**				
22	23.7	21.9	1.43	31.4
23	37.5	15.1	1.79	27
24	35.3	11.0	1.67	23.2
25	43.2	12.3	2.22	24.4
26	22.2	11.9	1.50	18.4
27	41.0	14.8	1.86	22.1
28	26.2	10.6	1.47	21.8
29	23.7	21.9	2.27	24

N – Newton, Nr – Number.

**Figure 1:**
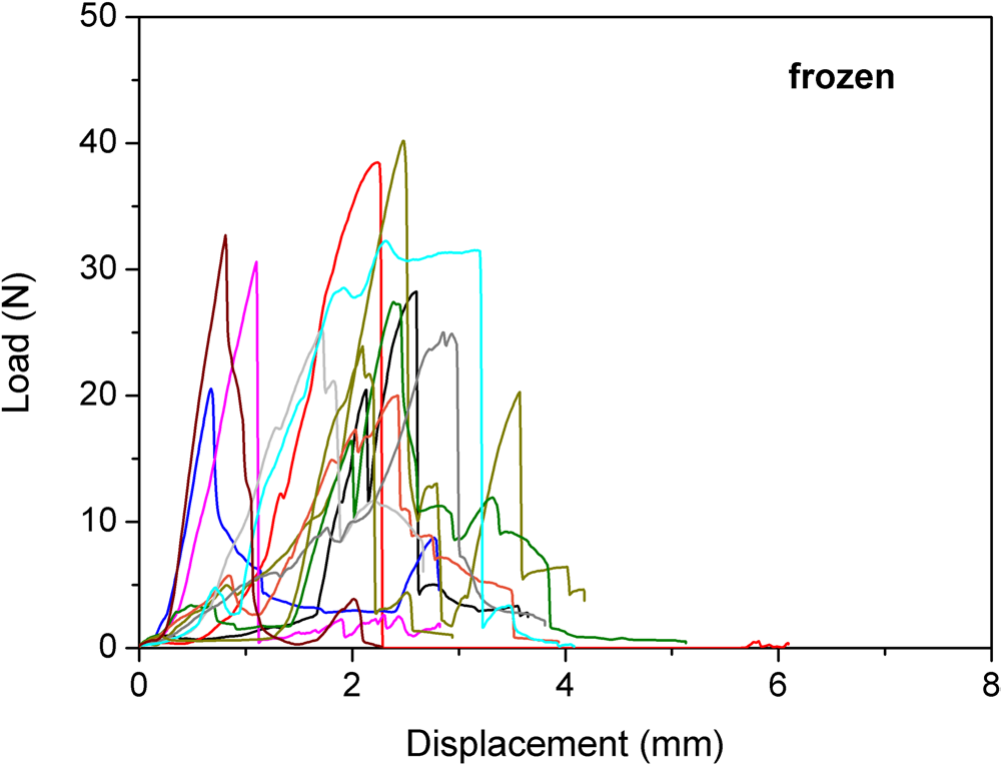
Behaviour of the frozen samples during load to failure test.

**Figure 2:**
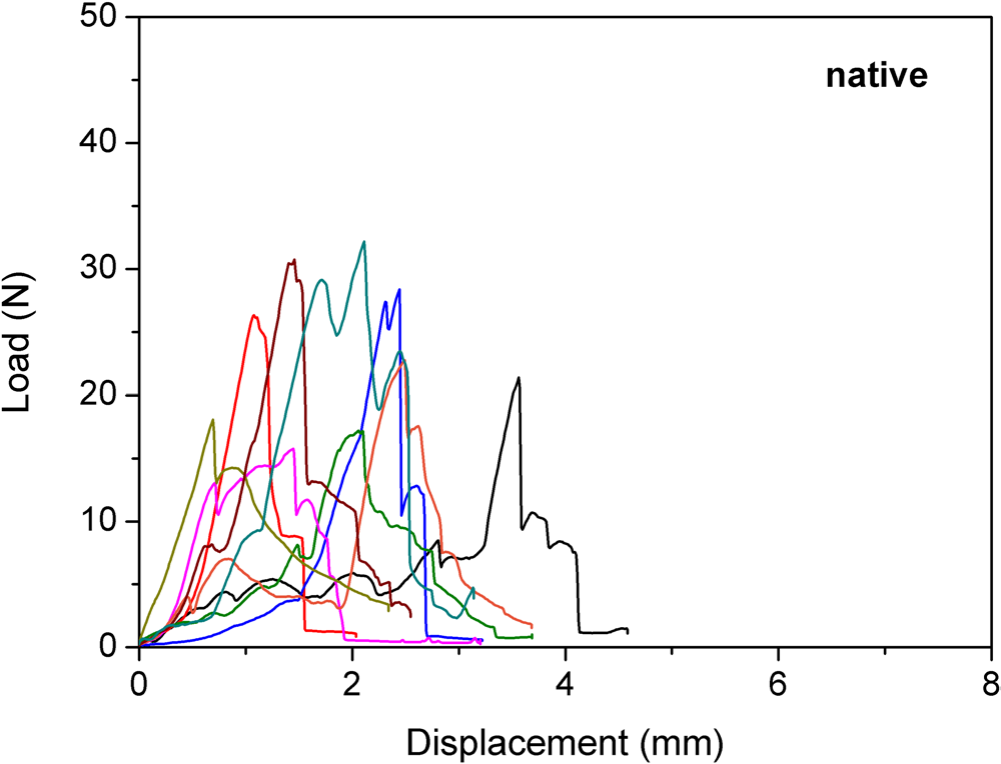
Behaviour of the paraformaldehyde fixed samples during load to failure test.

**Figure 3:**
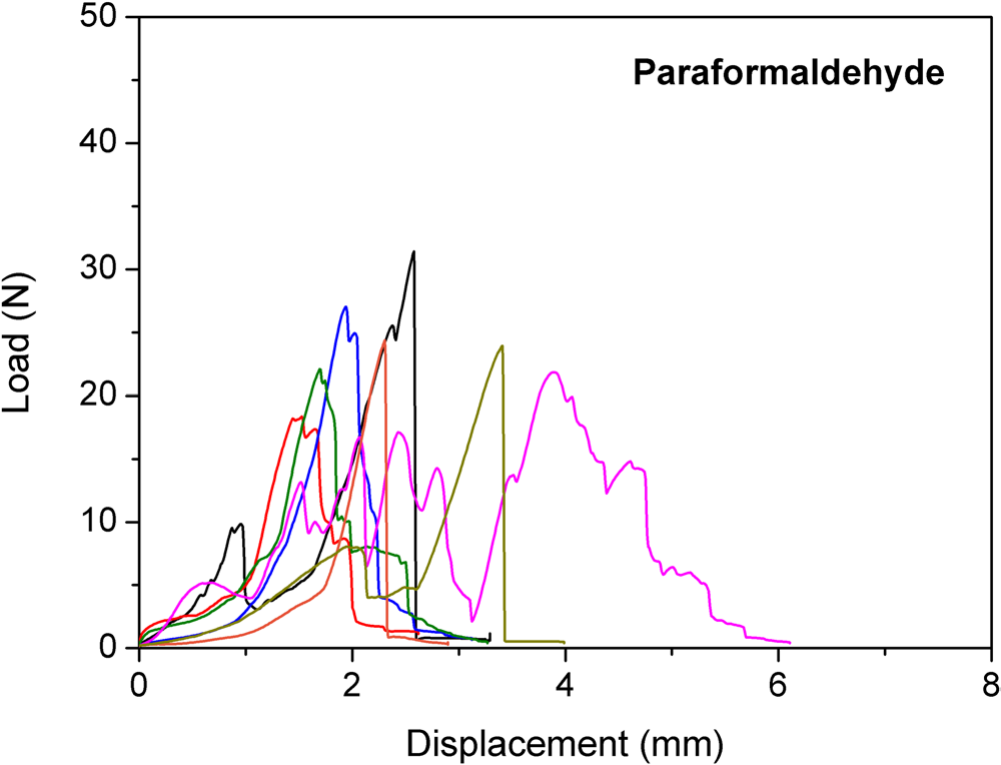
Behaviour of the native samples during load to failure test. Each curve represents one sample during the load to failure test. Starting at 0 N, load increases to maximum and then decreases.

**Figure 4:**
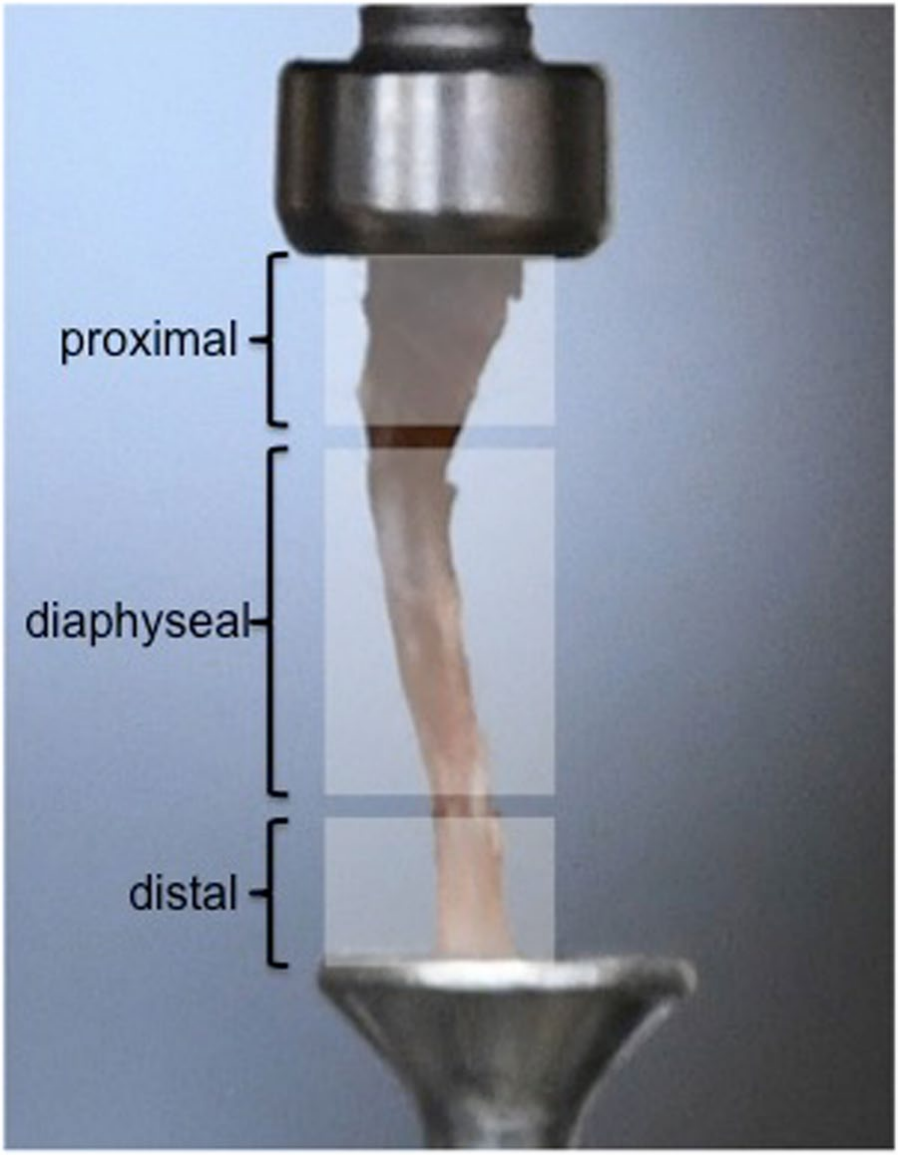
Definition of fracture location in detail.

No significant difference in the load to failure values between the three groups was found (p = 0.113). The mean load to failure in group 1 was 28.7 ± 6.1 N (median; 27.8 N; range 20 to 40.2 N) compared to 23.8 ± 3.8 N (median; 23.1 N; range; 18.4 to 31.4 N) in group 2. The mean load to failure in group 3 was 23.7 ± 5.7 N (median; 22.8; range 15.8 to 32.2 N). Details of load to failure are demonstrated in Figs 1, 2, and 3. Fractures occurred in the proximal, the diaphyseal as well as the distal regions of the tibia (Fig. 4).

In group 1, six fractures of the proximal, two of the diaphysis and four of the distal region were observed. In group 2, five fractures occurred in the proximal and four in the diaphyseal region. In group 3, one fracture occurred in the distal part and the remaining ones in the proximal part.

The highest load to failure values with a mean of 31.6 ± 6.3 N (median; 32.3; range 20.6 to 40.2 N) were registered in bones that broke in the distal region. The distal region of the tibia was able to withstand more load compared to the other parts of the tibia (mean 25 N proximal and medial region to 32 N in the distal region).

## Discussion

This study demonstrates for the first time that there are no significant differences in the mechanical properties of bone tissue after cryopreservation or paraformaldehyde fixation after short-term storage when comparing to the mechanical properties of native bone tissue.

The storage method of cryopreservation at −80 °C is simple and showed no changes in biomechanical testing, including stiffness and strength, when compared to the native samples. However, it needs to be mentioned that in this study the storage period was only 14 days, which is a relatively short period. Other studies report a storage period of over 6 months without impairments on biomechanics^11^. In fact, prolonged cryopreservation of 5 years of bone allografts is described without showing changes in biomechanical properties^12^. Therefore, this simple method seems to be ideal for biomechanical testing and long time storage.

Paraformaldehyde fixation is an alternative method that was evaluated in our study. The results of the load to failure, as well as stiffness and strength after paraformaldehyde fixation did not differ from the native bone. We consider this very valuable knowledge for planning future animal studies because it allows the use of bone tissue for further histological evaluation^13–15^. However, a recent study by Cheng P *et al*.^11^ reported negative effects on biomechanical properties of bone after long time storage using paraformaldehyde fixation. Our results, in contrast, did not show a negative influence of this method on the mechanical properties after short-term storage (2 weeks). We believe that this discrepancy might be due to different storage times between both studies.

We must therefore emphasize that paraformaldehyde and formalin based fixation methods may only be suitable for short-term storage since negative effects on biomechanical testing are mentioned even 2 weeks after storage^16,17^. Moreover, there is evidence showing that even the use of PBS to wash out ethanol or formaldehyde negatively affects biomechanical properties of bone tissue^18^.

A close look at the literature shows that formalin causes changes in the organic matrix with irreversible alteration of material and mechanical properties^9,10^. Even immediate impairment (after 12 h) of biomechanical properties is described when using formaldehyde^19^ or ethanol and therefore both substances are not recommended if material characteristics close to the *in-vivo* conditions are of interest^9^.

Based on the findings of this study, fixation with paraformaldehyde seems to be a suitable alternative when further histological investigation is planned and a short-term storage of up to 14 days can be ensured. We suggest that cryopreservation should be preferred if the focus is on the biomechanical properties of bone and a long storage time is intended.

## Limitations

Firstly, this study was limited to mouse bone tissue, only focusing on comparing load to failure between two different fixation methods with a storage period of only two weeks. However, this is one of the first studies comparing mechanical properties following −80C cryopreservation and paraformaldehyde fixation to fresh bone tissue.

Secondly, no evaluation of bone quality was made prior to the final load to failure test. However, same bone quality has been assumed due to the same age of the mice, the same race (C57BL/6) as well as the same holding and feeding conditions.

## Conclusion

This is the first study that directly compares two common preservation methods of bone tissue. We found that the investigated storage methods over a two-week period did not influence the biomechanical properties of murine bone tissue compared to native bone tissue. Thus, −80 °C cryopreservation as well as paraformaldehyde fixation seem to be equally suitable for biomechanical testing (load to failure) of the bone after a short-terms storage of up to 14 days.

## Material and Methods

### Animals

This study was performed as an anatomical specimen study at the Department of Trauma and Orthopaedic Surgery, Medical University of Vienna in cooperation with the Institute of Material Science and Technology, Vienna University of Technology. The study design was double blinded.

Fifteen 18-week-old female C57BL/6 mice were included in this study. For the surgical procedure all mice were anesthetized with 0.1 ml/10 g narcotic mix (ketamine 0.5 ml + 0.15 ml Rompun + 0.1 ml Dormicum in 5 ml NaCl) via subcutaneous injection with a 27-G needle. Directly postoperative 0.1 ml/10 g of glucose mix was injected. Enrofloxazin (7.5 mg/kg) was administered for infection prophylaxis pre-operatively as well as on the first post-operative day. For post-operative analgesic therapy the animals received Buprenorphin 0.1 mg/kg s.c. immediately after surgery (under general anaesthesia) and Piritramid 15 mg in 250 ml drinking water ad libitum together with 10 ml glucose (10%) for 5 days. The animals, 5 per group, were held in type 3 Makrolon cages at a temperature of 22 ± 2 °C, humidity of 55% ± 10%, a 12 h light cycle and they received food and water ad libitum.

Included animals were sacrificed with ketamine and heart puncture according to the guidelines of the Centre of Biomedical Research of the Medical University of Vienna.

Following death, both tibias were harvested and separated from the surrounding soft tissue and the fibula. The tibias were then randomly divided into three groups with different storage methods: Group 1 frozen at −80 °C (n = 12), Group 2 paraformaldehyde solution (n = 8) and Group 3 native group (n = 9). Each of the harvested tibias was individually stored in a tube.

### Storage of bone

In group 1 the bones were stored in a freezer at −80 °C. For testing, the bones were defrosted over 72 hours prior to testing in a refrigerator at 4 °C.

In group 2 the bones were put in a 4% paraformaldehyde working solution for 48 hours. After this period the bones were washed on a shaker with phosphate buffered saline (PBS) for another 48 hours. The PBS was changed after 24 hours. Specimens were then stored in the refrigerator at 4 °C until mechanical testing.

A storage time of two weeks prior to the experiment was chosen for group 1 and 2. In group 3, referred to as “native group”, the bones were not stored at all and tested immediately after harvesting.

### Biomechanical Testing

A researcher specialized in material science carried out the biomechanical testing. The graft link preparation and mechanical testing procedures were performed at the Institute of Material Science and Technology of the Technical University of Vienna.

**Figure 5:**
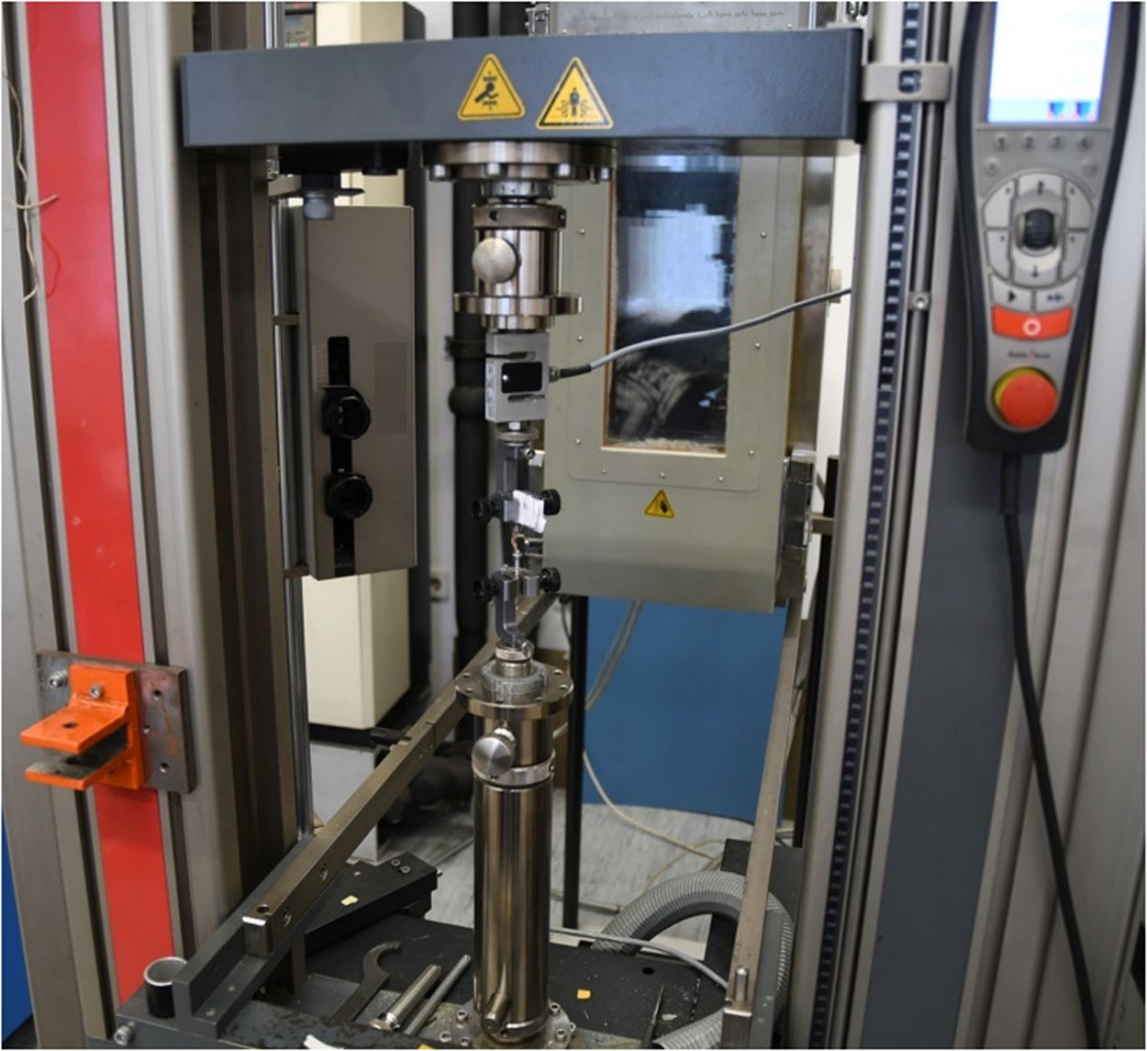
Testing method. Method used to test the bone with axial compression. The bone is attached to the materials testing system (Zwick Z050, Zwick Roell, Germany). General view presenting the testing machine with the moving crosshead at the top, the two clamps for fixation (with the bone sample in between) and the remote control.

A total of 29 out of 30 tibial bones underwent a standardized testing procedure. Each bone was mounted on a universal material testing machine (Zwick Z050, Fig. 5), equipped with a 100 Newton (N) load cell. For documentation purposes photographs were taken with a Nikon D500 digital single-lens reflex (DSLR) camera.

**Figure 6:**
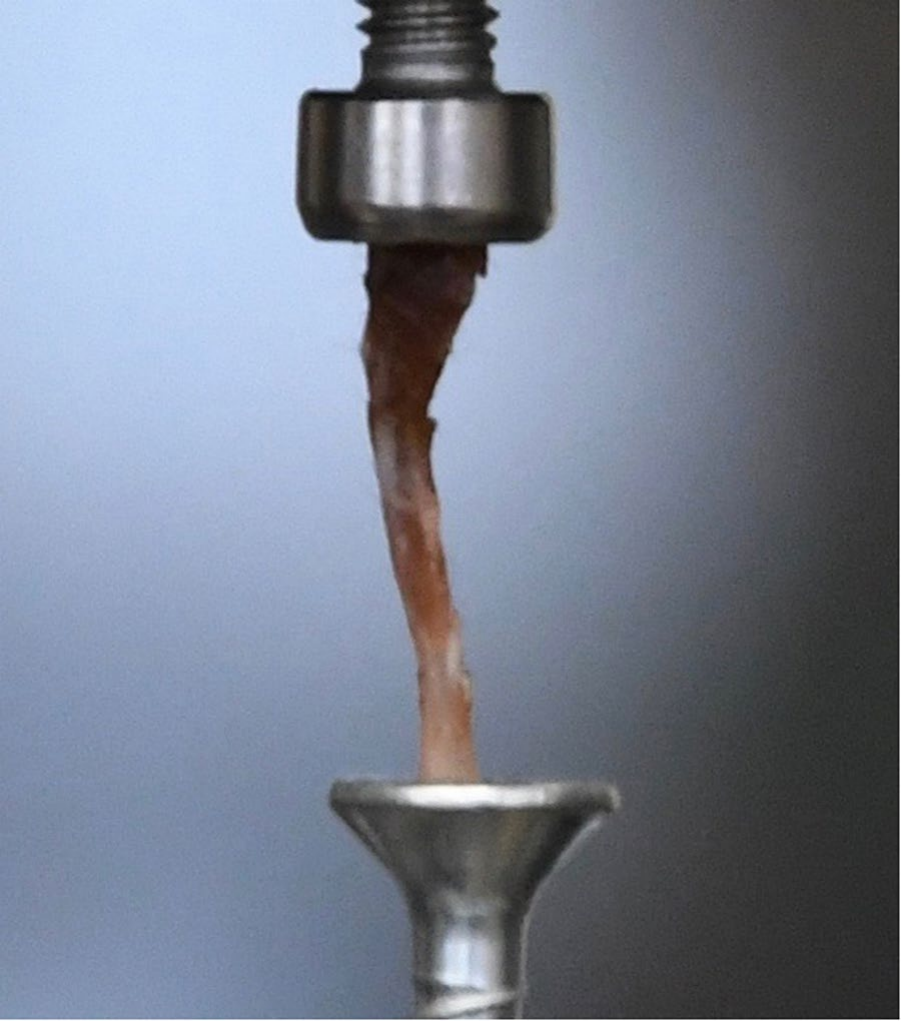
Close view of bone fixation during testing procedure.

The bones were fixed onto the adapter of the material testing system with two screws (Fig. 6) to perform axial compression on the bone.

Final testing was performed with a load to failure test at 10 millimetres per minute (standard procedure in literature)^20^.

In this study we focused on the load to failure values and on the anatomical region of the fracture. This was divided into the proximal part, the middle (diaphysis) and the distal part of the tibia.

### Statistics

A normal distribution of data was assumed, which was tested using the Shapiro-Wilk’s test. Homogeneity was evaluated with the Levene test. When both assumptions applied, a mixed-model ANOVA was used to test differences between the three groups. Descriptive statistics, including means and standard deviations, were performed for all three groups. Statistical significance was set at a level of p < 0.05.

All calculations were made using Microsoft Excel®, SPSS® software (Version 23.0, SPSS Inc., Chicago, IL, USA).

### Ethics

Prior to commencing the study the corresponding animal ethics review board (Ethik-Kommission der MUW zur Beratung und Begutachtung von Forschungsprojekten am Tier) and the BMFWF (Bundesministerium für Wissenschaft, Forschung und Wirtschaft) approved the study (ZI. 177/115-97/98 out 2014/15). The entire study was conducted according to the guidelines of the Medical University Vienna, Good Scientific Practice. Additionally the manuscript was written according the ARRIVE guidelines. Level of evidence is not applicable as this is a basic science research.

## Data Availability

The data that support the findings of this study are available from the corresponding author upon reasonable request.

## References

[1] Jepsen KJ, Silva MJ, Vashishth D, Guo XE, van der Meulen MC (2015). Establishing biomechanical mechanisms in mouse models: practical guidelines for systematically evaluating phenotypic changes in the diaphyses of long bones. Journal of bone and mineral research: the official journal of the American Society for Bone and Mineral Research.

[2] Fyhrie DP, Christiansen BA (2015). Bone Material Properties and Skeletal Fragility. Calcified tissue international.

[3] Nazarian A, Hermannsson BJ, Muller J, Zurakowski D, Snyder BD (2009). Effects of tissue preservation on murine bone mechanical properties. Journal of biomechanics.

[4] Brodt MD, Ellis CB, Silva MJ (1999). Growing C57Bl/6 mice increase whole bone mechanical properties by increasing geometric and material properties. Journal of bone and mineral research: the official journal of the American Society for Bone and Mineral Research.

[5] Berman AG, Clauser CA, Wunderlin C, Hammond MA, Wallace JM (2015). Structural and Mechanical Improvements to Bone Are Strain Dependent with Axial Compression of the Tibia in Female C57BL/6 Mice. PloS one.

[6] Sinder BP (2015). Rapidly growing Brtl/+ mouse model of osteogenesis imperfecta improves bone mass and strength with sclerostin antibody treatment. Bone.

[7] Linde F, Sorensen HC (1993). The effect of different storage methods on the mechanical properties of trabecular bone. Journal of biomechanics.

[8] Beaupied H (2006). The mode of bone conservation does not affect the architecture and the tensile properties of rat femurs. Bio-medical materials and engineering.

[9] Hammer N (2014). Ethanol and formaldehyde fixation irreversibly alter bones’ organic matrix. Journal of the mechanical behavior of biomedical materials.

[10] Morita K (2013). Influence of formalin fixation on the implant stability quotient and mechanical characteristics of bone. Br J Oral Maxillofac Surg.

[11] Cheng P (2016). Effects of Different Preservation Methods on Mechanical Properties of Mouse Femur. Sheng wu yi xue gong cheng xue za zhi = Journal of biomedical engineering = Shengwu yixue gongchengxue zazhi.

[12] Salai M, Brosh T, Keller N, Perelman M, Dudkiewitz I (2000). The effects of prolonged cryopreservation on the biomechanical properties of bone allografts: a microbiological, histological and mechanical study. Cell Tissue Bank.

[13] Frank JD, Balena R, Masarachia P, Seedor JG, Cartwright ME (1993). The effects of three different demineralization agents on osteopontin localization in adult rat bone using immunohistochemistry. Histochemistry.

[14] Schaepe K (2015). Assessment of different sample preparation routes for mass spectrometric monitoring and imaging of lipids in bone cells via ToF-SIMS. Biointerphases.

[15] Xin X (2018). Laser-Capture Microdissection and RNA Extraction from Perfusion-Fixed Cartilage and Bone Tissue from Mice Implanted with Human iPSC-Derived MSCs in a Calvarial Defect Model. Methods Mol Biol.

[16] Ohman C, Dall’Ara E, Baleani M, Van Sint Jan S, Viceconti M (2008). The effects of embalming using a 4% formalin solution on the compressive mechanical properties of human cortical bone. Clinical biomechanics (Bristol, Avon).

[17] Wieding J, Mick E, Wree A, Bader R (2015). Influence of three different preservative techniques on the mechanical properties of the ovine cortical bone. Acta of bioengineering and biomechanics.

[18] Vesper EO, Hammond MA, Allen MR, Wallace JM (2017). Even with rehydration, preservation in ethanol influences the mechanical properties of bone and how bone responds to experimental manipulation. Bone.

[19] Fiedler IAK, Casanova M, Keplinger T, Busse B, Muller R (2018). Effect of short-term formaldehyde fixation on Raman spectral parameters of bone quality. J Biomed Opt.

[20] Chon CS, Yun HS, Kim HS, Ko C (2017). Elastic Modulus of Osteoporotic Mouse Femur Based on Femoral Head Compression Test. Appl Bionics Biomech.

