# Author Correction: Effect of two (short-term) storage methods on load to failure testing of murine bone tissue

**DOI:** 10.1038/s41598-020-66764-6

**Published:** 2020-06-16

**Authors:** Thomas M. Tiefenboeck, Stephan Payr, Olga Bajenov, Thomas Koch, Micha Komjati, Kambiz Sarahrudi

**Affiliations:** 1 Department of Orthopaedics and Trauma Surgery, Division of Trauma Surgery, Medical University of Vienna, Vienna, Austria; 2 Institute of Materials Science and Technology, TU Wien, Vienna, Austria; 3 Department of Orthopaedics, Sacred Heart Hospital of Vienna, Vienna, Austria

Correction to: *Scientific Reports* 10.1038/s41598-019-42476-4, published online 11 April 2019

The original version of this Article contained errors.

In the Abstract,

“The comparison of the mean load to failure of all 3 groups (group 1: 28.7 N ± 6.1 N, group 2: 23.8 N ± 3.8 N and group 3: 23.7 N ± 5.7 N) did not reveal a significant difference.”

now reads:

“The comparison of the mean load to failure of all 3 groups (group 1: 28.7 N ± 6.4 N, group 2: 23.7 N ± 6.0 N and group 3: 24.0 N ± 3.9 N) did not reveal a significant difference.”

In Table 1,

“Stiffness in N/mm^2^”.

now reads:

“Stiffness in N/mm”

Furthermore, in column “Strength in N/mm^2^”, row r7,

“-27.1”

now reads:

“27.1”

Additionally Table 1 contained errors in the values in columns Stiffness in N/mm, Strength in N/mm², Cross sectional area in mm². The original Table 1 appears below.

**Table 1:** Details of stiffness, strength, Cross sectional area and load to failure of testes bones.

Nr. Group	Stiffness in N/mm^2^	Strength in N/mm^2^	Cross sectional area in mm^2^	Load to failure in N
**Freezing (n** = **12)**				
1	44.6	16.8	1.23	20.6
2	36.8	16.6	1.21	20
3	44.8	21.0	1.91	40.2
4	39.0	15.4	1.63	25
5	52.8	12.7	1.89	23.9
6	27.1	16.2	1.74	28.2
7	19.8	−27.1	1.13	30.6
8	23.5	15.1	1.67	25.2
9	28.3	19.1	1.43	27.4
10	27.2	12.6	2.57	32.3
11	42.6	18.3	1.79	32.7
12	63.4	20.9	1.84	38.5
**Native Bone (n** = **9)**				
13	49.6	11.3	2.52	28.4
14	44.3	10.4	1.52	15.8
15	29.3	10.6	2.01	21.4
16	39.7	12.0	2.66	18.1
17	32.0	23.1	1.33	30.7
18	48.2	12.1	2.69	17.2
19	24.9	13.3	1.72	22.8
20	38.4	18.7	1.41	26.3
21	46.3	13.9	2.32	32.2
**Paraformaldehyde (n** = **8)**				
22	23.7	21.9	1.43	31.4
23	37.5	15.1	1.79	27
24	35.3	11.0	1.67	23.2
25	43.2	12.3	2.22	24.4
26	22.2	11.9	1.50	18.4
27	41.0	14.8	1.86	22.1
28	26.2	10.6	1.47	21.8
29	23.7	21.9	2.27	24

N – Newton, Nr – Number.

As a result of this, in the Results section,

“The mean cross sectional area of ROI (fracture region) was 1.81 mm^2^ (median; 1.74 mm^2^ range; 1.13 to 2.69 mm^2^ STD 0.44 mm^2^). There was no significant difference between mean stiffness (freezing 37.5 N/mm^2^ vs. native 39.2 N/mm^2^ vs. 32.7 N/mm^2^ paraformaldehyde) and strength (freezing 17.6 N/mm^2^ vs. native 14.3 N/mm^2^ vs. 13.94 N/mm^2^ paraformaldehyde). A detailed overview is presented in Table 1.”

now reads:

“The mean cross sectional area of ROI (fracture region) was 1.72 mm^2^ (median; 1.67 mm^2^ range; 1.13 to 2.57 mm^2^ STD 0.38 mm^2^).There was no significant difference between mean stiffness (freezing 37.5 N/mm vs. native 39.2 N/mm vs. 32.7 N/mm paraformaldehyde) and strength (freezing 17.6 N/mm^2^ vs. native 13.9 N/mm^2^ vs. 13.9 N/mm^2^ paraformaldehyde). A detailed overview is presented in Table 1.”

And,

“No significant difference in the load to failure values between the three groups was found (p  =  0.113). The mean load to failure in group 1 was 28.7  ±  6.1 N (median; 27.8 N; range 20 to 40.2 N) compared to 23.8  ±  3.8 N (median; 23.1 N; range; 18.4 to 31.4 N) in group 2. The mean load to failure in group 3 was 23.7  ±  5.7 N (median; 22.8; range 15.8 to 32.2 N).”

now reads:

“No significant difference in the load to failure values between the three groups was found (p = 0.113). The mean load to failure in group 1 was 28.7 ± 6.4 N (median; 27.8 N; range 20 to 40.2 N) compared to 23.7 ± 6.0 N (median; 22.8 N; range; 15.8 to 32.2 N) in group 2. The mean load to failure in group 3 was 24.0 ± 3.9 N (median; 23.6; range 18.4 to 31.4 N).”

These errors have now been corrected in the PDF and HTML versions of the Article.

